# Pure 2D Perovskite Formation by Interfacial Engineering Yields a High Open‐Circuit Voltage beyond 1.28 V for 1.77‐eV Wide‐Bandgap Perovskite Solar Cells

**DOI:** 10.1002/advs.202203210

**Published:** 2022-11-13

**Authors:** Rui He, Zongjin Yi, Yi Luo, Jincheng Luo, Qi Wei, Huagui Lai, Hao Huang, Bingsuo Zou, Guangyao Cui, Wenwu Wang, Chuanxiao Xiao, Shengqiang Ren, Cong Chen, Changlei Wang, Guichuan Xing, Fan Fu, Dewei Zhao

**Affiliations:** ^1^ College of Materials Science and Engineering & Institute of New Energy and Low‐Carbon Technology Engineering Research Center of Alternative Energy Materials & Devices Ministry of Education Sichuan University Chengdu 610065 P. R. China; ^2^ Joint Key Laboratory of the Ministry of Education Institute of Applied Physics and Materials Engineering University of Macau Avenida da Universidade, Taipa Macau 999078 P. R. China; ^3^ Laboratory for Thin Films and Photovoltaics Empa – Swiss Federal Laboratories for Materials Science and Technology Ueberlandstrasse 129 Duebendorf CH‐8600 Switzerland; ^4^ Guangxi Key Laboratory of Processing for Non‐ferrous Metals and Featured Materials School of Resources, Environment and Materials Guangxi University Nanning 530004 P. R. China; ^5^ Ningbo Institute of Materials Technology and Engineering Chinese Academy of Sciences Ningbo New Material Testing and Evaluation Center Co., Ltd Ningbo City 315201 P. R. China; ^6^ School of Optoelectronic Science and Engineering & Collaborative Innovation Center of Suzhou Nano Science and Technology Key Lab of Advanced Optical Manufacturing Technologies of Jiangsu Province & Key Lab of Modern Optical Technologies of Education Ministry of China Soochow University Suzhou 215006 P. R. China

**Keywords:** interfacial passivation, organic arylammonium salts, perovskite solar cells, wide‐bandgap perovskites

## Abstract

Surface post‐treatment using ammonium halides effectively reduces large open‐circuit voltage (*V*_OC_) losses in bromine‐rich wide‐bandgap (WBG) perovskite solar cells (PSCs). However, the underlying mechanism still remains unclear and the device efficiency lags largely behind. Here, a facile strategy of precisely tailoring the phase purity of 2D perovskites on top of 3D WBG perovskite and realizing high device efficiency is reported. The transient absorption spectra, cross‐sectional confocal photoluminescence mapping, and cross‐sectional Kelvin probe force microscopy are combined to demonstrate optimal defect passivation effect and surface electric‐field of pure *n* = 1 2D perovskites formed atop 3D WBG perovskites via low‐temperature annealing. As a result, the inverted champion device with 1.77‐eV perovskite absorber achieves a high *V*_OC_ of 1.284 V and a power conversion efficiency (PCE) of 17.72%, delivering the smallest *V*_OC_ deficit of 0.486 V among WBG PSCs with a bandgap higher than 1.75 eV. This enables one to achieve a four‐terminal all‐perovskite tandem solar cell with a PCE exceeding 25% by combining with a 1.25‐eV low‐bandgap PSC.

## Graphical abstract

Tailoring *n* = 1 pure 2D perovskites on 3D perovskite surface via low‐temperature phenethylammonium bromide (PEABr) post‐treatment strongly suppresses the defects at the grain boundaries of 3D perovskites, which enables a high open‐circuit voltage of 1.284 V and a low open‐circuit voltage loss of 0.486 V for highly efficient inverted 1.77‐eV wide‐bandgap perovskite solar cells.

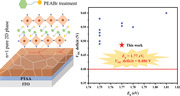

## 1 Introduction

Organic–inorganic hybrid perovskite solar cells (PSCs) have achieved great advances in power conversion efficiencies (PCEs) in the past decade.^[^ ^1^, ^2^ ^]^ Lab‐scale (≈0.1 cm^2^) single‐junction PSCs have reached a certified efficiency of 25.7%, on par with the record crystalline silicon (c‐Si) solar cells and approaching the Shockley–Queisser (S–Q) radiative efficiency limit.^[^ ^3^ ^]^ Constructing tandem solar cells (TSCs) is an effective way to surpass the single‐junction S–Q limit and realize higher efficiencies by fully utilizing the solar spectrum and reducing the thermalization losses of high‐energy photons.^[^ ^4^, ^5^, ^6^ ^]^

Owing to broad bandgap tunability and facile preparation process, perovskite‐based TSCs, which generally consist of a wide‐bandgap (WBG, ≈1.7–1.8 eV) perovskite top subcell absorbing high‐energy photons and a low‐bandgap (LBG) bottom subcell harvesting low‐energy photons such as c‐Si,^[^ ^7^, ^8^, ^9^, ^10^, ^11^ ^]^ copper indium gallium diselenide (CIGS), and mixed tin (Sn)‐lead (Pb) perovskite,^[^ ^12^, ^13^, ^14^, ^15^, ^16^, ^17^, ^18^, ^19^, ^20^, ^21^, ^22^, ^23^, ^24^ ^]^ have gained considerable interest. Hitherto, certified PCEs of 29.8%, 24.2%, and 26.4% have been achieved for two‐terminal perovskite/c‐Si, perovskite/CIGS, and perovskite/perovskite TSCs, respectively,^[^ ^25^ ^]^ offering great promise. However, the PCEs of perovskite‐based TSCs are still far below their theoretical efficiency limits,^[^ ^26^, ^27^ ^]^ which can be mainly ascribed to inferior performance of WBG PSCs due to defects in the bulk and on the surface of bromine (Br)‐rich perovskite absorbers,^[^ ^28^, ^29^ ^]^ leading to large open‐circuit voltage (*V*_OC_) deficits (defined as *E*_g_/*q* − *V*_OC_, where *q* is elementary charge). Moreover, photoinduced iodine (I)–Br phase segregation also impairs the stability of WBG perovskites, resulting in unsatisfactory operational device stability.^[^ ^30^, ^31^ ^]^

Great efforts such as compositional engineering, additive engineering, and interfacial engineering, have been devoted to improving film quality of WBG perovskites, passivating surface defects, and optimizing energy level alignment, leading to reduction of *V*_OC_ deficits and performance improvement of WBG devices.^[^ ^32^, ^33^, ^34^, ^35^, ^36^, ^37^, ^38^, ^39^, ^40^, ^41^, ^42^ ^]^ FA*_x_* Cs_1−_ *_x_* Pb(I*_y_* Br_1−_ *_y_*)_3_ (FA = formamidinium, Cs = cesium), the most commonly used WBG perovskite, was first developed by McMeekin et al. to achieve a PCE of around 17% with a *V*_OC_ of 1.2 V for 1.7‐eV n‐i‐p (normal structure) PSC.^[^ ^33^ ^]^ Yu et al. employed lead thiocyanate (Pb(SCN)_2_) additive and solvent annealing to enlarge the grain size of the perovskite film and decrease the defects at grain boundaries (GBs), realizing a *V*_OC_ of 1.25 V for 1.75‐eV n‐i‐p PSC.^[^ ^34^ ^]^ Chen et al. employed guanidinium bromide (GABr) on the perovskite surface to build graded perovskite homojunction, promoting the energy level matching and achieving a *V*_OC_ of 1.24 V for a 1.75‐eV p‐i‐n (inverted structure) device.^[^ ^35^ ^]^

Organic arylammonium salts, such as phenethylammonium (PEA) halide, have played tremendous roles in interfacial passivation and inhibition of ion migration for a wide range of perovskites.^[^ ^36^, ^43^, ^44^, ^45^, ^46^ ^]^ However, the underlying mechanisms are still under debate. On the one hand, the passivation effect on perovskites could originate from the direct deposition of organic arylammonium salts themselves, for example, phenethylammonium iodide (PEAI) and phenethylammonium bromide (PEABr).^[^ ^45^, ^47^ ^]^ On the other hand, low‐dimensional perovskites would form on the surface of 3D perovskites via the reaction of organic arylammonium salts with excess lead iodide (PbI_2_). It is widely accepted that the excess PbI_2_ is beneficial to passivate the defects at the GBs of perovskite,^[^ ^48^ ^]^ leading to improved initial efficiency. However, many recent studies showed that excess PbI_2_ is a critical source of intrinsic instability under illumination and can be detrimental to device stability.^[^ ^49^, ^50^, ^51^, ^52^, ^53^ ^]^ The *n* value (e.g., (R‐NH_3_)_2_A*_n_*_−1_M*_n_* X_3_ *_n_*_+1_ [*n* = 1, 2, 3, 4…] denotes Ruddlesden–Popper [RP] perovskites, where *n* refers to the number of the intermediate phase) of 2D perovskite can significantly affect carrier transport characteristics in PSCs. Westbrook et al. have demonstrated that the *n* value is critical for the charge injection from perovskite to charge transport layer. *n* = 1 is more favorable for the charge injection, however, at *n* > 1, the injection deteriorates. Furthermore, Zhang et al. have found that *n* = 1 2D perovskite atop 3D perovskite greatly reduces the severe interfacial nonradiative recombination.^[^ ^54^, ^55^ ^]^

Therefore, it is highly desired to investigate how one could control the phase purity of 2D perovskites on 3D perovskite bulk via post treatment of organic arylammonium salts and understand the in‐depth working mechanism. These insights would be beneficial to achieve high *V*_OC_ values and efficient p‐i‐n WBG PSCs, especially for the bandgaps ranging from 1.75 to 1.85 eV which are more suitable for fabrication of highly efficient all‐perovskite TSCs in combination with LBG perovskite subcells with the bandgaps generally ranging from 1.2 to 1.3 eV.^[^ ^6^, ^56^, ^57^ ^]^

Herein, we precisely tailor the phase purity of 2D perovskites on 3D perovskite surface by PEABr post‐treatment with different annealing temperatures, and systematically investigate the effects on the quality of both 1.77‐eV WBG perovskite bulk and surface. It is found that a pure *n* = 1 2D perovskite phase is preferably formed at a low temperature of 60 °C, showing a better passivation effect for surface defects and optimized surface electric field. Our best‐performing device achieves a high *V*_OC_ of 1.284 V and a PCE of 17.72% for a 1.77‐eV p‐i‐n WBG PSC, leading to a record‐low *V*_OC_ deficit of 0.486 V for WBG PSCs with a bandgap higher than 1.75 eV. Combined with a 1.25‐eV LBG PSC, we construct a four‐terminal (4‐T) all‐perovskite TSC and obtain a PCE of 25.17%. Our work provides new insights into PEABr post‐treatment on WBG perovskites and an effective approach to improve the performance of WBG devices and all‐perovskite TSCs.

## 2 Results and Discussion

**Figure 1:**
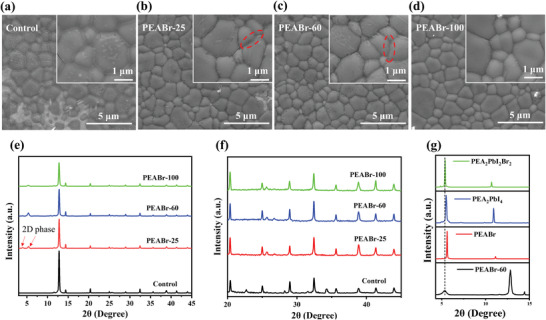
Top‐view SEM images of control and target perovskite films with PEABr post‐treatment at various temperatures: a) control, b) PEABr‐25, c) PEABr‐60, and d) PEABr‐100. The insets are magnified SEM images. XRD patterns of control and target perovskite films with PEABr post‐treatment at various temperatures: e) 2*θ* range from 3° to 45° and f) 2*θ* range from 20° to 45°. g) XRD patterns with peak position comparison of PEABr‐60, PEABr, PEA_2_PbI_4_, and PEA_2_PbI_2_Br_2_ perovskites.

We first investigated the effect of PEABr post‐treatment without and with different annealing temperatures of 25, 60, and 100 °C on a hotplate (denoted as control, PEABr‐25, PEABr‐60, and PEABr‐100, respectively) on the morphology of WBG perovskite films. **Figure** **1**a–d shows the top‐view scanning electron microscopy (SEM) images of the control and PEABr‐treated perovskite films. No obvious difference is observed in the distribution of grain size. However, some substances are found to accumulate at the GBs of the control film (inset of Figure 1a), which are considered as segregated PbI_2_ as reported.^[^ ^58^, ^59^ ^]^ After PEABr post‐treatment, the aggregates at the GBs disappear, and some lamellae emerge at the GBs for the PEABr‐25 and PEABr‐60 films (circled of Figure 1b,c). When the annealing temperature of PEABr is increased to 100 °C, the surface of the PEABr‐100 film becomes more uniform and smoother (Figure 1d). The lamellae located at the GBs vanish, and no apparent difference can be found between grains and GBs (inset of Figure 1d).

We speculate that the changes of the morphology after PEABr post‐treatment can be attributed to the chemical reaction between PEABr and PbI_2_ or the perovskite bulk. The PEABr molecule may react with PbI_2_ located at the GBs at low annealing temperature (e.g., 25 or 60 °C), resulting in formation of 2D PEA_2_Pb(I_1−_ *_x_* Br*_x_*)_4_ perovskites. Whereas, a higher annealing temperature (e.g., 100 °C) makes PEABr or PEA_2_Pb(I_1−_ *_x_* Br*_x_*)_4_ more readily react with 3D perovskite bulk to form (PEA)_2_(FA*_y_* Cs_1−_ *_y_*)*_n_*_−1_Pb*_n_* (I_1−_ *_x_* Br*_x_*)_3_ *_n_*_+1_.^[^ ^44^ ^]^

We conducted X‐ray photoelectron spectroscopy (XPS) measurements to inspect the reaction products after PEABr treatment. The peaks of Br 3d (Figure S1a, Supporting Information) and Pb 4f (Figure S1b, Supporting Information) shift toward lower binding energy after PEABr treatment. Compared with the control film, the PEABr‐25 and PEABr‐60 films show a slight shift (≈0.2 eV for both Br 3d and Pb 4f), while the PEABr‐100 film shows a large shift (≈0.8 and ≈0.6 eV for Pb 4f and Br 3d, respectively). However, the positions of the N 1s and Cs 3d peaks after PEABr treatment do not change much (Figure S1c,d, Supporting Information). This indicates that PEABr deposited on the perovskite surface interacts with PbI_2_ located at GBs at a low annealing temperature, and further interplays with the octahedral [PbX_6_]^4−^ (X = I, Br) from 3D perovskite bulk at a higher annealing temperature, which results in more intense interaction and obvious shift of binding energy for Pb 4f and Br 3d.

We then measured X‐ray diffraction (XRD) patterns of control and PEABr treated films with different annealing temperatures to examine their structural and chemical properties, as shown in Figure 1e. All samples exhibit a strong PbI_2_ peak located at 12.8°, and show negligible difference on crystallinity (Figure 1f), consistent with the SEM results. While, PEABr treatment reduces the intensity of PbI_2_ peak, but introduces additional weak peaks at 3.9° and 5.3° for the PEABr‐25 film and merely a peak at 5.3° for the PEABr‐60 and PEABr‐100 films. To further explore the origin of these peaks, we measured the XRD patterns of the PEABr, PEA_2_PbI_4_, and PEA_2_PbI_2_Br_2_ films (Figure 1g). It is interesting that both PEABr‐60 and PEA_2_PbI_2_Br_2_ films exhibit an identical diffraction peak at 5.3°. The difference in full width half maximum between PEABr‐60 sample and pure 2D PEA_2_PbI_2_Br_2_ perovskite is probably due to the different formation process. Moreover, we also directly deposited PEABr on the as‐prepared PbI_2_ film, followed by thermal annealing process at 60 °C, as well as comparing the XRD patterns of the *n* = 1 PEA_2_PbI_4−_ *_x_* Br*_x_* 2D perovskites with *x* = 1, 2, and 3 (Figure S2, Supporting Information). It is confirmed that the peak at 5.3° originates from 2D RP PEA_2_PbI_2_Br_2_ perovskite (*n* = 1) rather than PEABr or another PEA_2_PbI_4−_ *_x_* Br*_x_* complex. For the PEABr‐25 film, the peak located at 3.9° may correspond to *n* = 2 quasi‐2D phase as reported previously.^[^ ^44^, ^60^ ^]^ More importantly, the intensity of *n* = 1 2D phase for the PEABr‐60 film is higher than that for the PEABr‐100 film, which further proves our hypothesis that a higher annealing temperature promotes the reaction of PEABr with 3D perovskites to form quasi‐2D large‐*n* (PEA)_2_(FA*_y_* Cs_1−_ *_y_*)*_n_*_−1_Pb*_n_* (I_1−_ *_x_* Br*_x_*)_3_ *_n_*_+1_ perovskites. Compared with that of 3D perovskites, the XRD peak of quasi‐2D large‐*n* (PEA)_2_(FA*_y_* Cs_1−_ *_y_*)*_n_*_−1_Pb*_n_* (I_1−_ *_x_* Br*_x_*)_3_ *_n_*_+1_ perovskites cannot be detected, probably due to the trace amount of quasi‐2D large‐*n* (PEA)_2_(FA*_y_* Cs_1−_ *_y_*)*_n_*_−1_Pb*_n_* (I_1−_ *_x_* Br*_x_*)_3_ *_n_*_+1_ perovskites.

**Figure 2:**
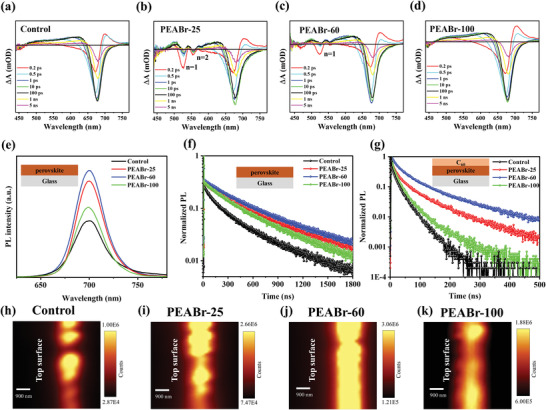
TA spectra of a) control, b) PEABr‐25, c) PEABr‐60, and d) PEABr‐100 perovskite films at different delay times. e) Steady‐state PL and f) TRPL spectra of WBG perovskite films with different post‐treatment process. g) TRPL spectra of WBG perovskite films with C_60_ coated on top. h–k) Cross‐sectional confocal PL mapping images of h) control, i) PEABr‐25, j) PEABr‐60, and k) PEABr‐100 WBG devices.

To further investigate the effect of post‐annealing temperature on the formation of 2D perovskites atop the surface of 3D WBG perovskites, we conducted transient absorption (TA) spectra to qualitatively analyze the composition of perovskite phase. **Figure** **2**a–d shows the TA spectra of the WBG perovskite films with and without PEABr treatment measured at different delay time. Compared with the control and PEABr‐100 films, the PEABr‐25 film shows two emerging photobleaching peaks at lower wavelengths, which are regarded as 2D perovskite phases (*n* = 1 and *n* = 2).^[^ ^60^, ^61^ ^]^ As the annealing temperature rises to 60 °C, the *n* = 2 2D perovskite phase disappears but the pure n = 1 2D perovskite phase still exists, which is in good agreement with our XRD results.^[^ ^60^, ^61^ ^]^ Further, when the annealing temperature increases to 100 °C, the 2D perovskite phase cannot be detected any more, and the corresponding spectra exhibit similar results to that of the control film, which is consistent with the intensity change of XRD peaks located at 5.3°. Such a trend has also been observed in the ultraviolet photoelectron spectroscopy (UPS) results (Figure S3, Supporting Information). Thus, we expect that more of pure 2D perovskites with low *n* value could better passivate the GBs of WBG perovskites.^[^ ^54^, ^55^ ^]^

We measured steady‐state photoluminescence (PL) and time‐resolved photoluminescence (TRPL) spectra to evaluate the carrier dynamics of these PEABr‐treated films (deposited on bare glass), as shown in Figure 2e,f. All the films with PEABr post‐treatment exhibit enhanced PL intensity at the wavelength of 700 nm which is consistent with the absorption spectra (Figure S4, Supporting Information), and the intensity reaches the highest when the annealing temperature is 60 °C, suggesting that the *n* = 1 2D PEA_2_PbI_2_Br_2_ perovskites can effectively decrease the defect density, and thus the nonradiative recombination in the PEABr‐60 film is greatly suppressed. However, the PL intensity of the PEABr‐100 film dramatically decreases, which may be ascribed to inferior effect of quasi‐2D large‐*n* (PEA)_2_(FA*_y_* Cs_1−_ *_y_*)*_n_*_−1_Pb*_n_* (I_1−_ *_x_* Br*_x_*)_3_ *_n_*_+1_ perovskites on the defect passivation. Moreover, the PEABr‐60 film shows a prolonged carrier lifetime of 659.18 ns, longer than those of the control (449.46 ns), PEABr‐25 (622.12 ns), and PEABr‐100 (551.75 ns) films, as shown in Table S1, Supporting Information. These results suggest that the pure *n* = 1 2D PEA_2_PbI_2_Br_2_ perovskites could effectively passivate the surface defects of WBG perovskite films.

To gain more insights into the interfacial recombination at the perovskite/fullerene (C_60_) interface, we deposited 20‐nm C_60_ on top of the perovskite and measured their TRPL decays shown in Figure 2g and Table S2, Supporting Information. A similar trend on carrier lifetime of these films is obtained, indicating PEABr treatment with low annealing temperature effectively inhibits the carrier recombination while high annealing temperature weakens this effect.

We also monitored the phase photostability of these perovskite films under continuous illumination (Figure S5, Supporting Information). The control film shows serious phase segregation, while the target films with PEABr treatment have significantly alleviated phase segregation. It is worth mentioning that the phase segregation does not happen to the PEABr‐60 film, implying that the pure 2D *n* = 1 PEA_2_PbI_2_Br_2_ perovskites stabilize the 3D perovskites with superior phase stability. Previous studies have pointed out that the light‐induced phase segregation in Br‐rich WBG perovskite likely originates from the ion migration, especially the diffusion and exchange of mobile ions at GBs and at the surface of perovskite.^[^ ^36^, ^62^ ^]^ 2D perovskites on top of the 3D perovskites is supposed to inhibit the ion migration via creating a surface barrier at the perovskite surface, preventing the diffusion of halide ions from the 3D perovskite film to the adjacent charge transport layer,^[^ ^63^, ^64^ ^]^ and thus suppressing the light‐induced phase segregation.

Surface treatment may improve not only the quality of perovskite surface but also the quality of perovskite bulk or buried underneath interface.^[^ ^65^, ^66^ ^]^ We conducted the cross‐sectional confocal PL mapping measurement to understand the depth‐dependent defect manipulation in these perovskite films with PEABr treatment, as shown in Figure 2h–k. Obviously, the control film displays a discontinuous luminescence, which may result from numerous defects at GBs. Owing to better passivation of 2D perovskite phases (*n* = 1 and *n* = 2), the PEABr‐25 and PEABr‐60 films exhibit enhanced luminescence intensity. Especially for the PEABr‐60 film, the luminescence of the top surface becomes more uniform and smoother, which implies effective suppression of nonradiative recombination due to the formation of pure 2D perovskites PEA_2_PbI_2_Br_2_ at 60 °C. Nevertheless, the PEABr‐100 film shows some relatively dark area through the cross section, suggesting an unsatisfactory defect passivation effect.

We performed atomic force microscopy (AFM) and Kelvin probe force microscopy (KPFM) measurements to further assess surface morphology and potential on WBG perovskites treated with PEABr. PEABr treatment first reduces the root‐mean‐square roughness (*R*_q_) from 18.7 nm for control film and 17.0 nm for PEABr‐25 film to 15.9 nm for PEABr‐60 film, and again increases *R*_q_ to 18.5 nm (Figure S6, Supporting Information). Figure S7a–h, Supporting Information, shows the KPFM potential mapping and KPFM line scans of control and PEABr‐treated perovskite films. The control film shows a relatively broader range of contact potential difference (CPD). When applying PEABr post‐treatment without thermal process (25 °C), the CPD at GBs and grain interiors (GIs) is reduced. After an annealing process of 60 °C, the CPD between GBs and GIs cannot be clearly distinguished. However, when the temperature is further raised to 100 °C, the CPD difference is enlarged again. We attribute these to superior passivation effect of pure 2D perovskites PEA_2_PbI_2_Br_2_ formed in and on top of PEABr‐60 film, which strongly reduces the defect density, possibly induced by lower defect formation energy at GBs.^[^ ^36^ ^]^ On the contrary, mixed quasi‐2D large‐*n* (PEA)_2_(FA*_y_* Cs_1−_ *_y_*)*_n_*_−1_Pb*_n_* (I_1−_ *_x_* Br*_x_*)_3_ *_n_*_+1_ perovskites render inferior ability in passivating GBs compared with PEABr‐25 and PEABr‐60 film with 2D‐preferred perovskite phases (*n* = 1 and *n* = 2).

**Figure 3:**
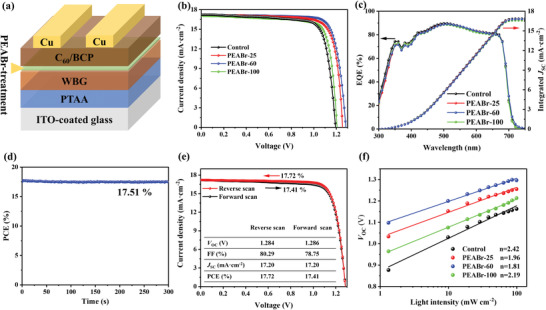
a) Schematic of the WBG PSC structure, b) *J*–*V* curves, and c) EQE spectra of the control and target devices with different post‐treatment process. d) Steady‐state efficiency of the device by tracking the maximum power point. e) *J*–*V* curves of the best‐performing PEABr‐60 device under forward and reverse voltage scans. f) Dependence of the *V*_OC_ on light intensity.

We fabricated the WBG PSCs with a structure of glass/indium tin oxide (ITO)/poly(bis(4‐phenyl)(2,4,6‐trimethylphenyl)amine) (PTAA)/WBG perovskite/C_60_/bathocuproine (BCP)/Cu, where WBG perovskite was post‐treated by PEABr or not (**Figure** **3**a). Their corresponding statistical photovoltaic parameters are shown in Figure S8 and Table S3, Supporting Information. The PEABr‐25 devices exhibit an enhanced average *V*_OC_ of 1.250 V compared with the control devices (1.182 V), while PEABr treatment at 60 °C further improves the average *V*_OC_ to a value as high as 1.277 V, together with an increased fill factor (FF) of 78.91%. However, high temperature treatment at 100 °C likely inducing large‐*n* quasi‐2D perovskites (PEA)_2_(FA*_y_* Cs_1−_ *_y_*)*_n_*_−1_Pb*_n_* (I_1−_ *_x_* Br*_x_*)_3_ *_n_*_+1_ as aforementioned attenuates passivation effect, leading to a decreased average *V*_OC_ of 1.204 V and FF of 77.15% and thus lower PCEs.

Figure 3b shows the current density‐voltage (*J*–*V*) curves of the best‐performing devices for the control and target devices, and the detailed photovoltaic parameters are summarized in Table S3, Supporting Information. The control device shows a PCE of 16.06% with a *V*_OC_ of 1.186 V, while the optimal PEABr‐60 device achieves the highest PCE of 17.72% and a remarkably enhanced *V*_OC_ of 1.284 V and a high FF of 80.29%, indicating a significantly reduced *V*_OC_ deficit less than 490 mV based on our 1.77 eV‐bandgap perovskite absorber, primarily originating from the passivation effect of pure 2D perovskites. Given that the S–Q *V*_OC_ limit could be calculated using a simple equation q*V*_OC_ = 0.941*E*_g_ − 0.171 eV,^[^ ^67^ ^]^ our champion device shows much reduced gap of 211 mV from the S–Q limit. To the best of our knowledge, these achievements are among the best values for inverted WBG PSC with a bandgap ranging from 1.75 to 1.80 eV for application in all‐perovskite TSCs to date (Table S4 and Figure S12, Supporting Information). The external quantum efficiency (EQE) spectra and the integrated short‐circuit current densities (*J*_SC_s) for these devices, as shown in Figure 3c, reflect no much difference in photocurrents, well consistent with the *J*_SC_s acquired from the *J*–*V* measurements. A steady‐state PCE of 17.51% of the PEABr‐60 PSC is obtained under the duration of 300 s maximum power point (MPP) tracking (Figure 3d). Our best‐performing device also exhibits negligible *J–V* hysteresis (Figure 3e and the inserted table).

We conducted light intensity‐dependent *J*–*V* measurements to examine charge recombination behavior in our control and target group. As shown in Figure 3f, a monotonic increase of *V*_OC_ with logarithmic light intensity is observed. By fitting the following equation:

(1) $$V_{\mathrm{OC}}=\mathrm{nkT}\ln\left(\phi_{\mathrm{light}}\right)/q$$

where *n*, *k*, *T*, *Φ*_light_, and *q* represents ideality factor, Boltzmann constant, absolute temperature, illumination intensity, and elementary charge, respectively. The PEABr‐60 device has the smallest *n* value of 1.81 among other relatively larger *n* values of 2.42, 1.96, and 2.19 for control, PEABr‐25, and PEABr‐100, respectively. This confirms that PEABr‐60 process effectively suppresses the trap‐assisted Shockley–Read–Hall recombination.^[^ ^16^ ^]^

To further assess the passivation effect of PEABr treatment with varying annealing temperature, we carried out transient photovoltage (TPV) and electrochemical impedance spectroscopy (EIS) measurements. As shown in Figure S9a, Supporting Information, the recombination lifetime of PEABr‐60 device is 13.7 µs, which is slightly higher than those of the control (8.7 µs), PEABr‐25 (10.8 µs), and PEABr‐100 (9.7 µs) devices, representing significantly hindered charge recombination in PEABr‐60 device, in good agreement with our TRPL results. EIS results further reveal the transport mechanism and charge recombination behavior of the WBG devices. Figure S9b, Supporting Information, shows the Nyquist plots of the control and target groups measured at a bias voltage of 0.9 V with sweep frequency ranging from 10 MHz to 10 Hz under dark condition. Devices with PEABr treatment through different processes all exhibit larger recombination resistance (*R*_rec_) at a low‐frequency region and smaller series recombination (*R*_s_) at high‐frequency stage. However, the PEABr‐60 device apparently shows the largest *R*_rec_ and smallest *R*_s_ among all the devices, implying restrained charge recombination and promoted charge transfer.^[^ ^68^ ^]^

**Figure 4:**
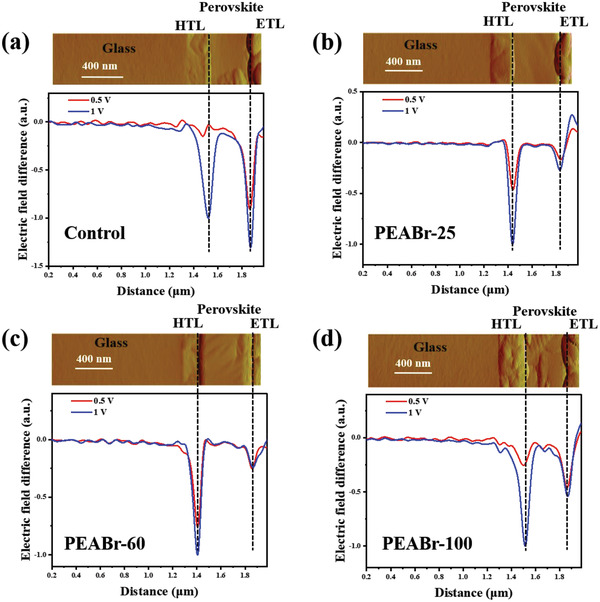
Cross‐sectional KPFM results: AFM maps and the corresponding electric field profiles of a) control, b) PEABr‐25, c) PEABr‐60, and d) PEABr‐100 PSCs.

We then employed cross‐sectional KPFM measurement to probe the electric‐field distribution across the entire device and gain more insights into the working mechanism of PEABr treatment at different annealing temperatures. **Figure** **4**a–d shows the cross‐sectional KPFM mappings and the electric‐field difference across the devices under different bias voltage with respect to 0 V bias. While, the corresponding potential‐profiling results and potential difference are shown in Figure S10, Supporting Information. The electric‐field difference exhibits two large peaks of equivalent intensity at the hole transport layer (HTL)/perovskite interface and the perovskite/electron transport layer (ETL) interface in our control group, in which the former intensity is lower than the latter one. However, after employing PEABr on the perovskite surface and applying a relatively low temperature, the peaks of electric‐field difference at the perovskite/ETL interface are significantly reduced while the peaks at the HTL/perovskite maintain high intensity. These results suggest that the main junction is located at the front side (i.e., HTL/perovskite interface) of the device. Since the PEABr treatment was applied on the perovskite top surface, we assume that the improvement is mainly at perovskite/ETL interface; hence we normalized the peak at HTL/perovskite interface to better compare the change at perovskite/ETL interface. During the measurement, the potential drop and the strength of the electric‐field difference across the device are determined by the competition between HTL/perovskite and perovskite/ETL interfaces, because the electric current through the whole device stack must be even.^[^ ^69^, ^70^, ^71^ ^]^ In our case, the main junction is changed to the HTL/perovskite interface while the perovskite/ETL interface could be assigned as “back‐contact.” The larger the peak at perovskite/ETL interface, the larger the leakage current. PEABr treatment with low‐temperature annealing process promotes the “back‐contact” quality and decreases the leakage current, which may be attributed to suppressed defect density. However, further increase in annealing temperature to 100 °C greatly reduces this effect (Figure 4f).

We monitored the device stability to estimate the impact of PEABr treatment. Figure S11a, Supporting Information, exhibits the MPP tracking of encapsulated PEABr‐60 and control devices measured in the air under white LED illumination. The PEABr‐60 one maintained 90% of its initial PCE after 103 h continuous illumination, while the control one lost 25% of its initial PCE only in 21 h. For the devices stored in N_2_‐filled glovebox, the PEABr‐60 device retained 97.3% PCE in 2 months and the control device only remained 58.3%. These results indicated that PEABr treatment can significantly enhance the device stability.

**Figure 5:**
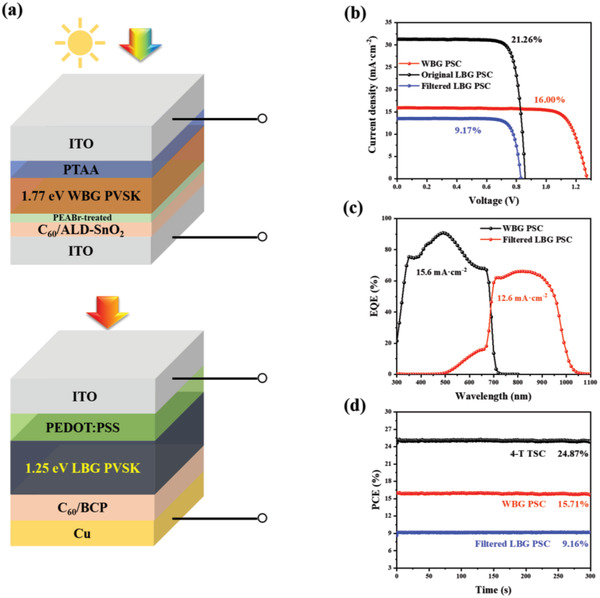
a) Schematic device configuration of a 4‐T all‐perovskite tandem cell. b) *J*–*V* curves, c) EQE spectra, and d) power outputs of WBG top cell, filtered LBG bottom cell, and the overall output under continuous AM 1.5G illumination for 300 s.

We then stacked a 4‐T all‐perovskite TSC  with the device structure shown in **Figure** **5**a. To protect the WBG perovskite film and C_60_ layer from the damage caused by sputtering ITO transparent electrode, we applied atomic layer deposited SnO_2_ (ALD‐SnO_2_) to replace BCP. The detailed photovoltaic parameters of semi‐transparent WBG, filtered LBG, and 4‐T all‐perovskite tandem devices are shown in Figure 5b–d and Table S5, Supporting Information. Benefitting from a high *V*_OC_ of 1.274 V with a high FF of 78.8% for PEABr‐treated semi‐transparent WBG device, our 4‐T tandem device achieves a high PCE of 25.17%, which is among the highest PCEs for 4‐T all‐perovskite TSCs.

## 3 Conclusion

We have demonstrated and unlocked the effect of PEABr post‐treatment on WBG perovskite bulk and surface as well as the device performance by systematically optimizing the annealing temperature of PEABr treatment. PEABr treatment at low‐temperature (60 °C) annealing forms a pure *n* = 1 2D perovskite phase on the WBG perovskite surface, which effectively passivates the surface defects and optimizes surface electric field, leading to significantly suppressed nonradiative recombination and improved perovskite/ETL “back‐contact” quality. As a result, an enhanced PCE of 17.72% with a highly improved *V*_OC_ exceeding 1.28 V has been obtained in inverted devices, which are the highest *V*_OC_ and PCE values among WBG PSCs with a bandgap higher than 1.75 eV to date. A 4‐T all‐perovskite TSC achieves a PCE of 25.17% by combining this semi‐transparent WBG device with a 1.25‐eV LBG device.

## 4 Experimental Section

### Materials

*N*,*N*‐dimethylformamide (DMF), dimethyl sulfoxide (DMSO), chlorobenzene, isopropanol (IPA), SnI_2_, SnF_2_, and Pb(SCN)_2_ were purchased from Sigma‐Aldrich. Diethyl ether was purchased from Chengdu Chron Chemical Co., Ltd., PbI_2_ and lead bromide (PbBr_2_) were purchased from TCI. Formamidinium iodide (FAI) and methylamine iodide (MAI) was purchased from Greatcell Solar Company. Cesium iodide (CsI) was purchased from Alfa Aesar. PTAA was purchased from Xi'an Polymer Light Technology Corporation. PEDOT:PSS (CLEVIOS P VP AI 4083) was purchased from Heraeus, LLC. PEABr was purchased from Youxuan technology company, C_60_ was purchased from Nano‐C, BCP was purchased from Jilin OLED, and copper (Cu) was purchased from Zhongnuoxincai Co., Ltd.

### Perovskite Precursor Preparation

The WBG perovskite FA_0.8_Cs_0.2_Pb(I_0.6_Br_0.4_)_3_ precursor was prepared by dissolving the 0.96 mmol FAI, 0.24 mmol CsI, 0.48 mmol PbI_2_, 0.72 mmol PbBr_2_, and 0.015 mmol Pb(SCN)_2_ in 1 mL DMF:DMSO (3:1/v:v) and stirred at 60 °C overnight. The LBG perovskite (FASnI_3_)_0.6_(MAPbI_3_)_0.4_ precursor was prepared by mixing the FASnI_3_ and MAPbI_3_ precursor with a stoichiometric ratio of 0.6:0.4 (v:v). The FASnI_3_ precursor solution was prepared by^[^ ^51^, ^52^ ^]^ dissolving 372 mg of SnI_2_, 3.9 mg SnF_2_, and 172 mg of FAI in mixed DMF and DMSO. The MAPbI_3_ precursor solution was prepared by dissolving 461 mg PbI_2_ and 159 mg MAI with 11.3 mg Pb(SCN)_2_ dissolved in 630 µL DMF and 70 µL DMSO.

### WBG PSCs Fabrication

Glass substrates coated with ITO (10 Ω sq^−1^) were ultrasonically cleaned with detergent, deionized water, and ethanol for 15 min in sequence. The ITO glass substrates were dried with nitrogen flow and a treatment with ultraviolet –ozone for 15 min was conducted before the deposition of HTL. The ITO/glass substrates were transferred to a glove box filled with nitrogen. PTAA dissolved in chlorobenzene (2 mg mL^−1^) was spin‐coated onto the ITO substrate at 4000 rpm for 30 s, followed by annealing at 100 °C for 10 min. Before the deposition of perovskite absorber, 60 µL DMF was spin‐coated on PTAA at 4000 rpm for 10 s to enhance the wettability. Then 70 µL precursor was dropped on PTAA and quickly spin‐coated through a two‐step process, that is, 500 rpm for 2 s and 4000 rpm for 60 s, and the antisolvent of 700 µL diethyl ether was dripped at 25 s of the second step. Then the as‐prepared perovskite film was annealed at 60 °C for 2 min and 100 °C for 10 min. For target group, 80 µL PEABr dissolved in IPA (2 mg mL^−1^) was spin‐coated onto the perovskite surface at 3000 rpm for 30 s after the as‐prepared perovskite films were cooled down to room temperature, and followed by annealing at 100, 60 °C, or without thermal process for PEABr‐100, PEABr‐60, and PEABr‐25 samples, respectively. For the post‐treatment annealing process, the temperature of hotplate was set at 25, 60, and 100 °C. To accurately control the temperature, all temperatures on the hotplate were calibrated by a thermocouple thermometer. All the samples were transferred to multisource evaporation chamber and 20 nm C_60_, 5 nm BCP, and 100 nm Cu were sequentially evaporated at 5 × 10^−4^ Pa. The area of solar cells was defined by the overlapping of metal electrode and patterned ITO. For the semi‐transparent WBG devices, ALD‐SnO_2_ was deposited after evaporating the C_60_ to replace the BCP. For the deposition of ALD‐SnO_2_, the chamber temperature was set at 100 °C, and the source temperature of tetrakis(dimethylamino) tin(iv) (TDMASn) and deionized water were set at 60 and 25 °C, respectively. The pulse time and purge time were 25 ms and 15 s for both TDMASn and water sources. And then 150 nm ITO was sputtered as transparent electrode. The pure 2D PEA_2_PbI_2_Br_2_ perovskite was grown by directly depositing the precursor (prepared with a stoichiometric ratio) on the bare glass and followed by a thermal annealing process. However, the 2D phase in PEABr‐60 sample was probably due to the reaction of PbI_2_ with PEABr.

### LBG PSCs Fabrication

The preparation process of ITO substrates was the same as the WBG PSC. PEDOT:PSS films were coated on the cleaned ITO substrate at 4000 rpm for 50 s and then dried at 145 °C for 45 min. The LBG perovskite precursor was spin‐coated onto ITO/PEDOT:PSS at 5000 rpm for 60 s. Diethyl ether was applied drop‐wise at 5 s onto the spinning substrate during the spin‐coating. All perovskite films were annealed at 70 °C for 3 min and then 105 °C for 7 min in a glovebox. Then, the perovskite thin films were transferred into a vacuum chamber for thermal evaporation of electron transport layers and metal contact, which were the same as WBG PSC. The active areas of the devices through an aperture mask were 0.0784 cm^2^. Devices were encapsulated with cover glass and UV‐curable epoxy.

### Film and Device Characterization

WBG perovskites with various post‐treatment were characterized by XRD, (Bruker D2 Phaser) with Cu‐K*α* (*λ* = 0.154 nm) radiation at 30 kV and 10 mA excitation. SEM images were taken with FE‐SEM, Regulus‐8230, Hitachi. Absorbance spectra of perovskite films were measured by UV–vis spectrophotometer (PerkinElmer Lambda 950). PL and TRPL were performed by FLS980 (Edinburgh Inc.) PL measurements were carried out using a 532‐nm Xeon lamp with a monochromator and TRPL were measured using a supercontinuum pulsed laser (Wuhan Yangtze Soton Laser Co. Ltd.) with a wavelength of 532 nm. Confocal PL was measured by WITec alpha300R. By controlling the focusing of the objective lens on the samples, the detector could vertically collect the spectral signals of samples layer by layer. Finally, the software was used to process these data and present the cross‐sectional confocal PL mapping. TA absorption measurements were performed using a Helios setup. The transient dynamics in fs–ns time region (50 fs–7 ns) were acquired by Helios that works in a nondegenerate pump‐probe configuration. 800 nm wavelength laser pulses were obtained from the regenerative amplifier's output while 400 nm wavelength laser pulses were obtained with a BBO doubling crystal. The probe pulses were a white light continuum generated by passing the 800 nm fs pulses through a sapphire plate for the visible part. Surface AFM and KPFM were taken with Bruker Nano Inc. DI Multi Mode 8 while cross‐sectional KPFM were taken by y a Veeco D5000 AFM equipped with a Nanoscope V controller. All the KPFM related measurements were conducted in an N_2_‐filled glove box. XPS and UPS were measured using a photoelectron spectrometer (ESCALAB 250Xi, Thermo Fisher Scientific). *J*–*V* curves were measured using Keysight B2901A source meter under AM1.5G (100 mW cm^−2^) illumination (Enlitech, SS‐F5) in an N_2_‐filled glove box. All *J*–*V* measurements were performed via an aperture with an active area of 0.0784 cm^2^. The spectral response was recorded by a solar cell quantum efficiency measurement system (QE‐R, Enlitech). TPV and EIS were measured by the all‐in‐one characterization system (PAIOS, Fluxim AG). The MPP tracking was measured by white LED simulator (Weina Bonuo new materials Co., Ltd., Qingdao) equivalent to AM1.5G, 100 mW cm^−2^.

### Statistical Analysis

For easy comparison, TRPL (Figure 1d), XPS spectra (Figure S11, Supporting Information), absorption spectra (Figure S4, Supporting Information), TPV decay (Figure S9, Supporting Information), and stability test (Figure S11, Supporting Information) were normalized. Originlab software was used for the statistical analysis of average values and the variances of PCE, *V*_OC_, FF, and *J*_SC_ parameters in Table S3, Supporting Information, and the corresponding statistical distribution box charts in Figure S8, Supporting Information. These statistical data were obtained from 20 cells for control and target groups. The average lifetime of TRPL characterizations in Tables S1 and S2, Supporting Information, was calculated via the equation $\tau_{\mathrm{avg}}=\left(A_{1}{\tau_{1}}^{2}+A_{2}{\tau_{2}}^{2}\right)/\left(A_{1}\tau_{1}+A_{2}\tau_{2}\right)$. The data for the rest of the charts were directly obtained during the measurements.

## Conflict of Interest

The authors declare no conflict of interest.

## Supporting information

Supporting Information

## Data Availability

The data that support the findings of this study are available from the corresponding author upon reasonable request.
