# Supplementary material for: Pure 2D Perovskite Formation by Interfacial Engineering Yields a High Open‐Circuit Voltage beyond 1.28 V for 1.77‐eV Wide‐Bandgap Perovskite Solar Cells
Source: Adv Sci (Weinh). 2022 Nov 13;9(36):2203210. doi: 10.1002/advs.202203210 (PMC9799022; doi:10.1002/advs.202203210)
Supplement: Supplementary file 1 — Supporting Information [file ADVS-9-2203210-s001.pdf]

ADVANCED SCIENCE
Open Access

Supporting Information

for *Adv. Sci.*, DOI 10.1002/advs.202203210

Pure 2D Perovskite Formation by Interfacial Engineering Yields a High Open-Circuit Voltage beyond 1.28 V for 1.77-eV Wide-Bandgap Perovskite Solar Cells

Rui He, Zongjin Yi, Yi Luo, Jincheng Luo, Qi Wei, Huagui Lai, Hao Huang, Bingsuo Zou, Guangyao Cui, Wenwu Wang, Chuanxiao Xiao, Shengqiang Ren, Cong Chen, Changlei Wang, Guichuan Xing, Fan Fu and Dewei Zhao**

Pure 2D Perovskite Formation by Interfacial Engineering Yields a High Open-circuit Voltage beyond 1.28 V for 1.77-eV Wide-bandgap Perovskite Solar Cells

Rui He, Zongjin Yi, Yi Luo, Jincheng Luo, Qi Wei, Huagui Lai, Hao Huang, Bingsuo Zou, Guangyao Cui, Wenwu Wang, Chuanxiao Xiao, Shengqiang Ren, Cong Chen Changlei Wang, Guichuan Xing, Fan Fu, Dewei Zhao**

R. He, Z. Yi, Y. Luo, J. Luo, G. Cui, Dr. W. Wang, Dr. S. Ren, Dr. C. Chen, Prof. D. Zhao

College of Materials Science and Engineering & Institute of New Energy and Low-Carbon Technology

Engineering Research Center of Alternative Energy Materials & Devices, Ministry of Education

Sichuan University

Chengdu 610065, China

E-mail: chen.cong@scu.edu.cn, dewei.zhao@scu.edu.cn, dewei_zhao@hotmail.com

Q. Wei, Prof. G. Xing

Joint Key Laboratory of the Ministry of Education

Institute of Applied Physics and Materials Engineering

University of Macau

Avenida da Universidade, Taipa, Macau 999078, China

H. Lai, Dr. F. Fu

Laboratory for Thin Films and Photovoltaics

Empa – Swiss Federal Laboratories for Materials Science and Technology

Ueberlandstrasse 129, CH-8600 Duebendorf, Switzerland

Dr. H. Hao, Prof. B. Zou

Guangxi Key Laboratory of Processing for Non-ferrous Metals and Featured Materials, School of Resources, Environment and Materials

Guangxi University, Nanning 530004, China.

Dr. C. Xiao

Ningbo Institute of Materials Technology and Engineering, Chinese Academy of Sciences
Ningbo New Material Testing and Evaluation Center CO., Ltd
Ningbo City 315201, China
Prof. C. Wang
School of Optoelectronic Science and Engineering & Collaborative Innovation Center of Suzhou Nano Science and Technology
Key Lab of Advanced Optical Manufacturing Technologies of Jiangsu Province & Key Lab of Modern Optical Technologies of Education Ministry of China
Soochow University, Suzhou 215006, China

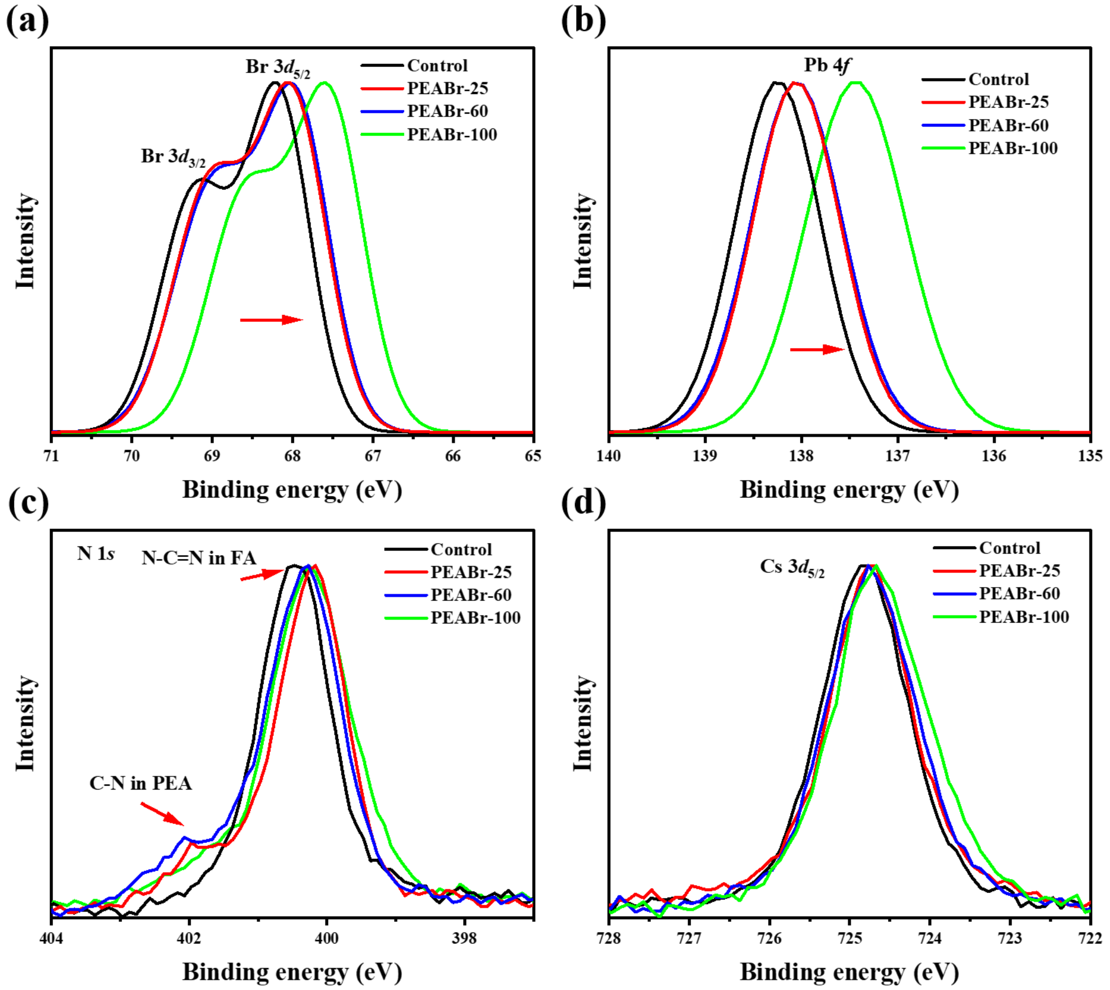

Figure S1. XPS spectra of a) Br $3d_{3/2}$ and Br $3d_{5/2}$, b) Pb 4f, c) N 1s, and d) Cs $3d_{5/2}$.

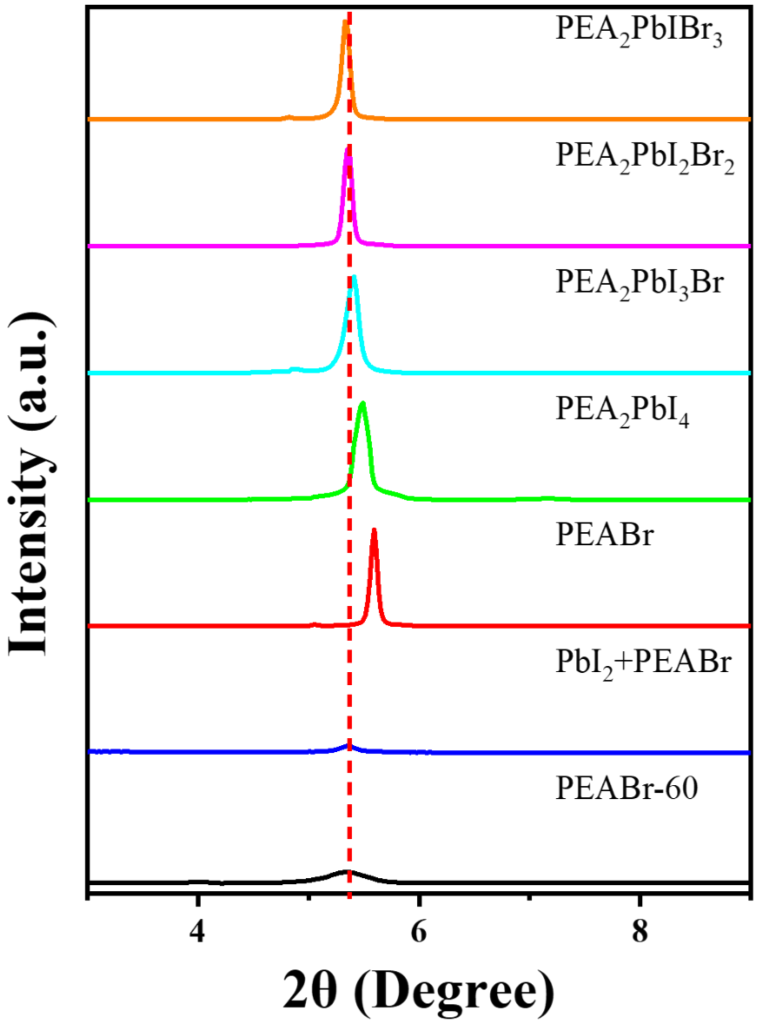

Figure S2. XRD patterns of $PEA_2PbI_{4-x}Br_x$, PEABr-60, and PbI_2+PEABr samples.

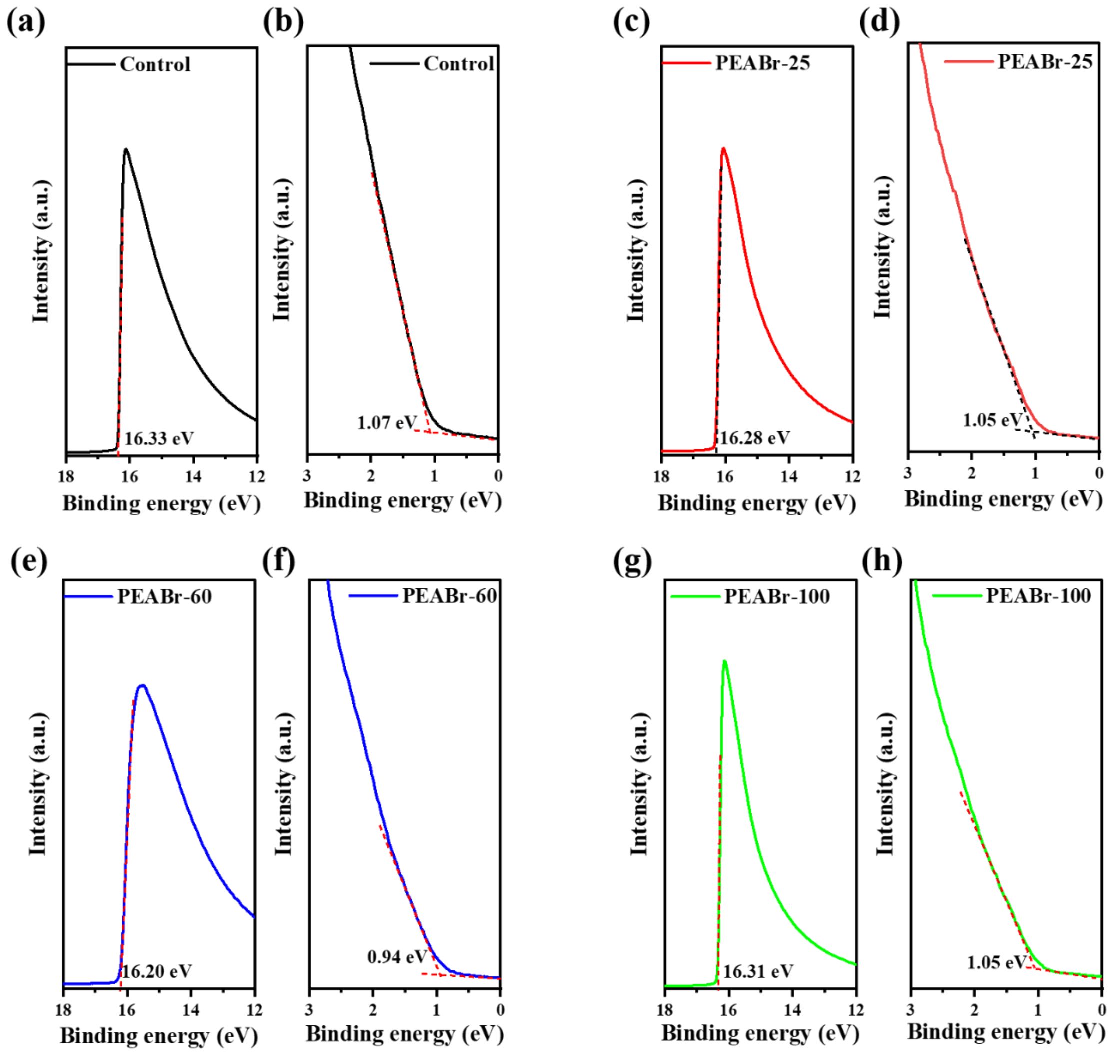

Figure S3. a, c, e, g) Secondary electron cut-off and b, d, f, h) valence band of control and target perovskite films treated with different process. According to Einstein's photo-emission law and calculating from the formula $E_F = h\nu - E_{cutoff}$, in which the hν is photon energy (21.22 eV here) and the E_{cutoff} represents the secondary electron cut-off edge.

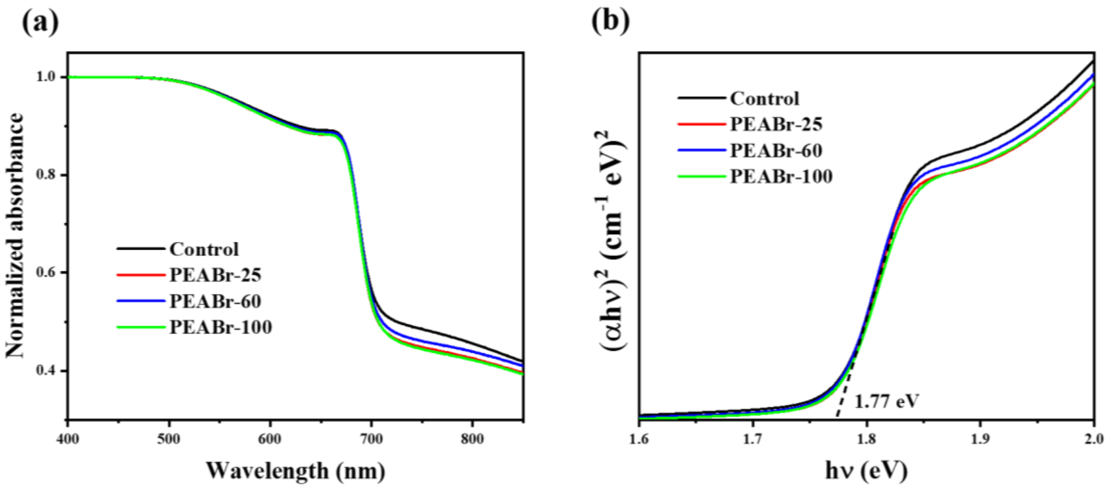

Figure S4. a) Absorbance spectra and b) Tauc plots of control and target perovskite films. The bandgap is obtained via Tauc plots according to the relation $(\alpha h\nu)^2 \propto h\nu - E_g$, where α is the absorption coefficient, resulting from the absorbance spectra and film thickness, h is Planck constant, ν is frequency. According to the Tauc plot, $(\alpha h\nu)^2$ is then plotted as a function of energy hν and the linear region is fitted, so that the bandgap results from an extrapolation of this linear fit to the x-axis.[1]

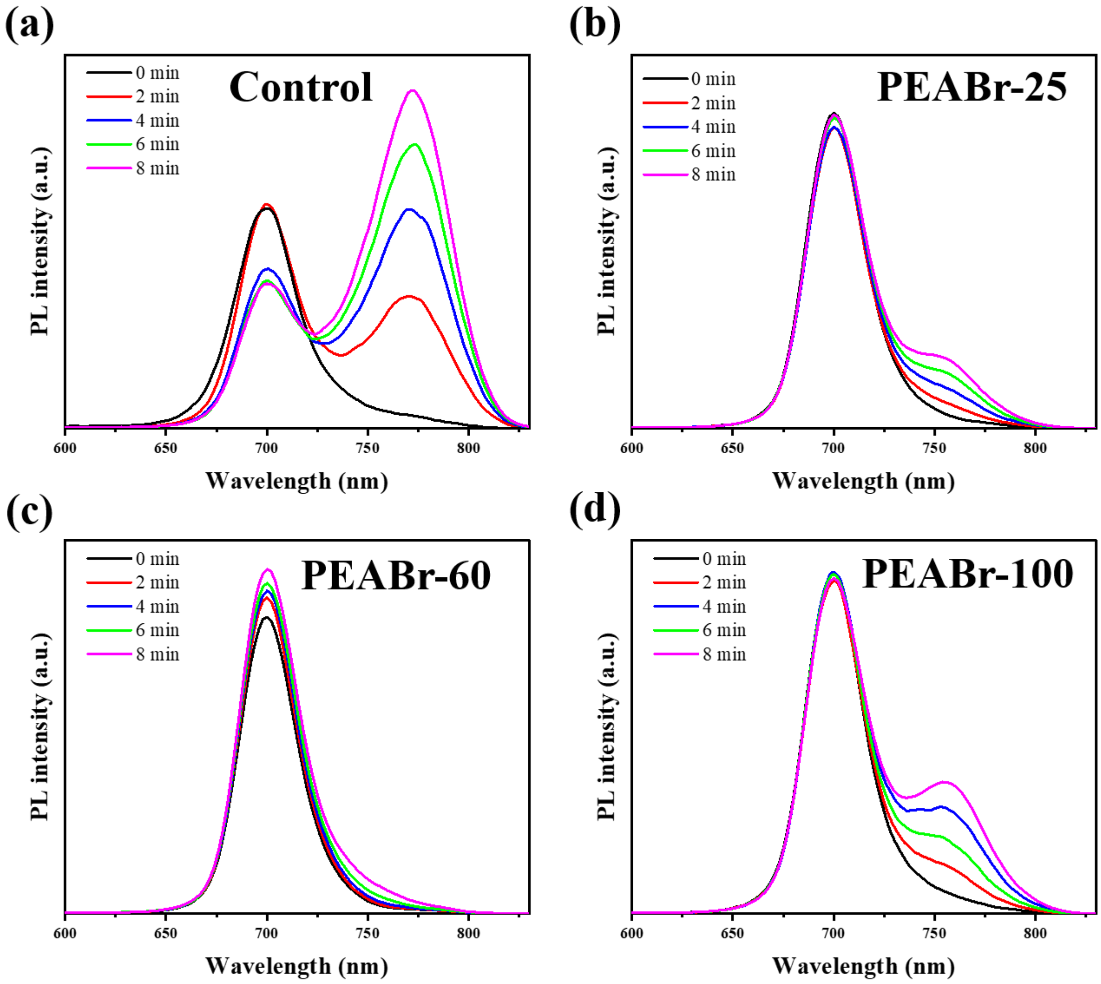

Figure S5. PL spectra of a) control, b) PEABr-25, c) PEABr-60, and d) PEABr-100 perovskite films under different illumination time.

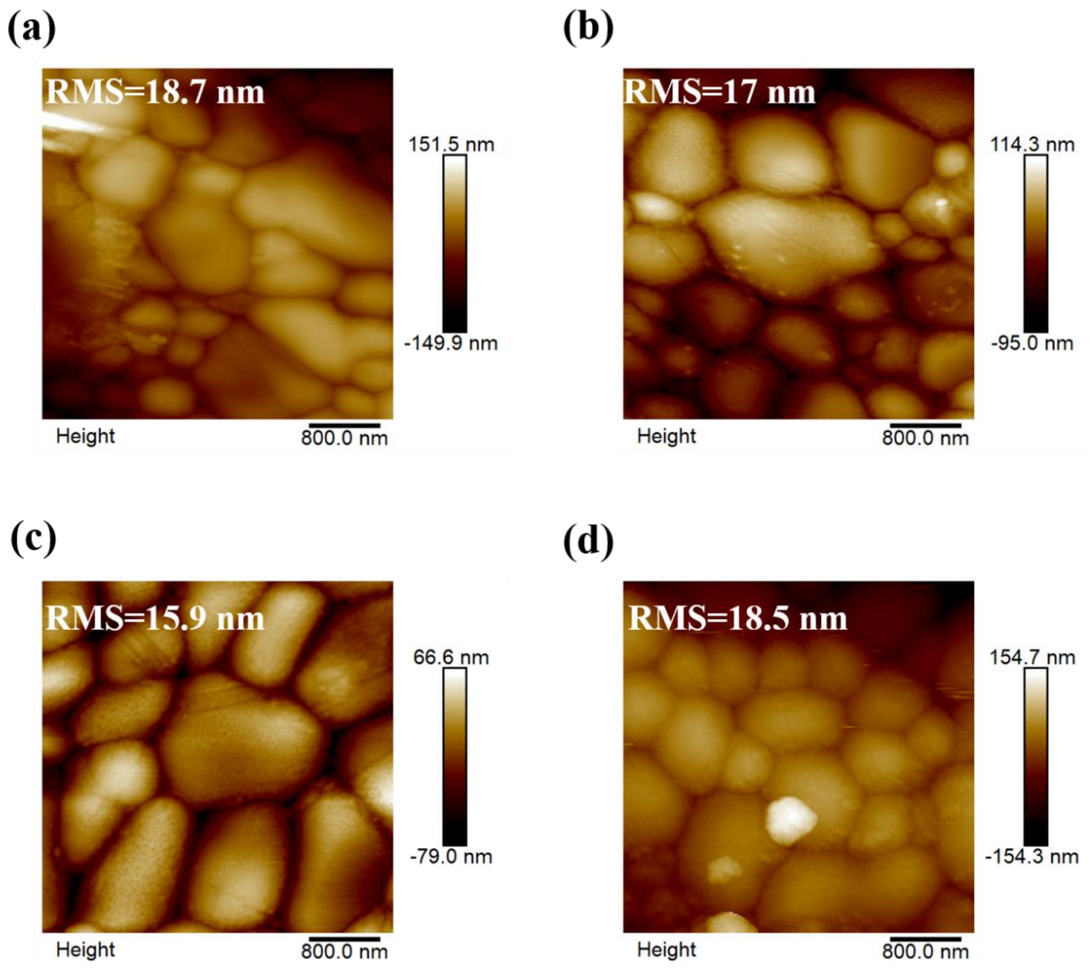

Figure S6. AFM images of a) control, b) PEABr-25, c) PEABr-60, and d) PEABr-100 perovskite films.

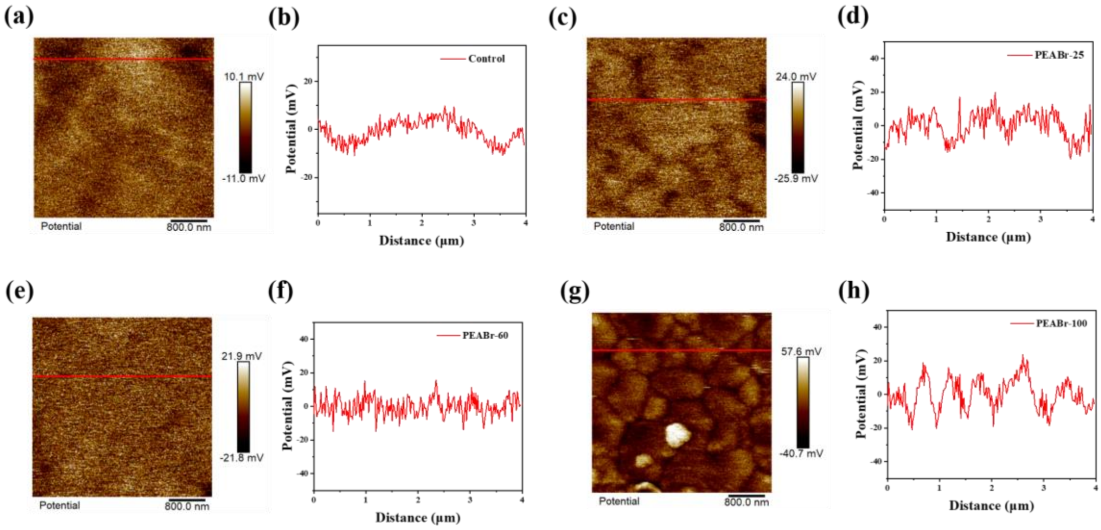

Figure S7. KPFM surface potential maps and corresponding potential line profiles of a, b) control, c, d) PEABr-25, e, f) PEABr-60, and g, h) PEABr-100 perovskite films.

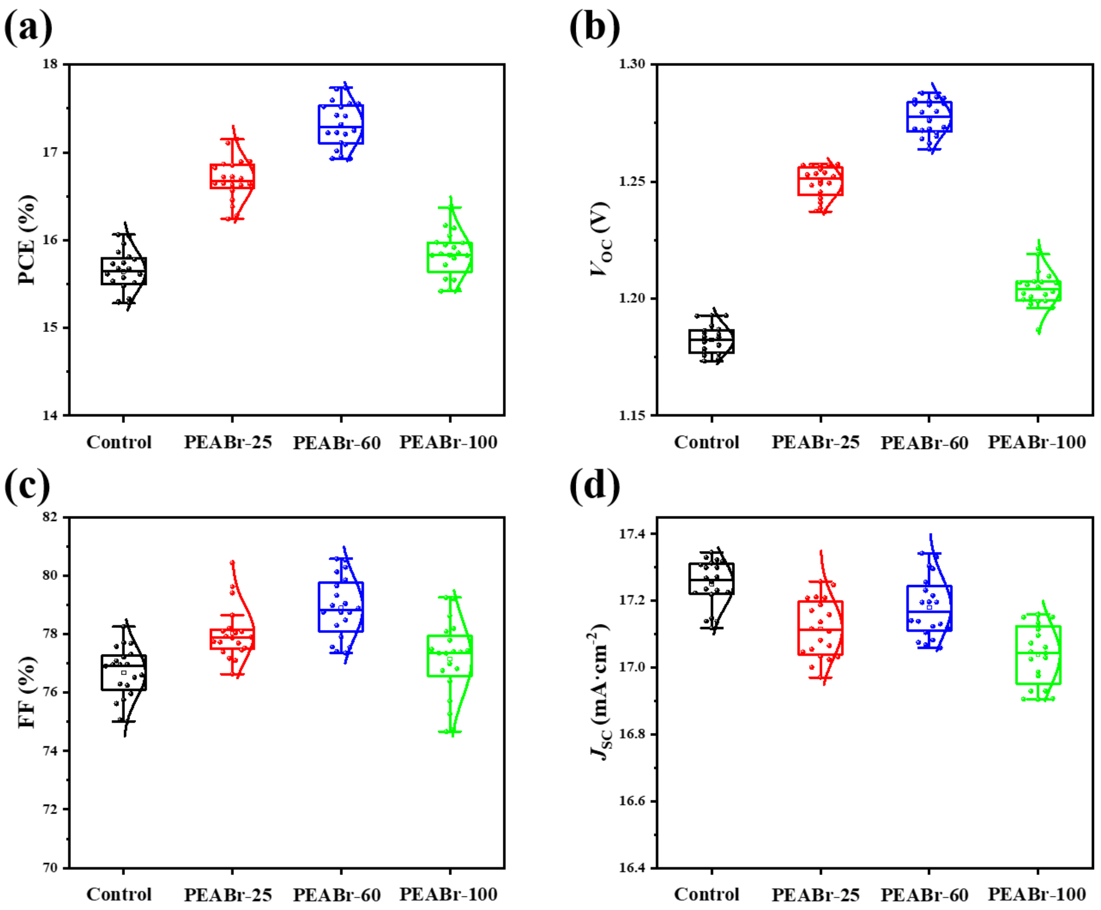

Figure S8. Box charts of a) PCE, b) V_{OC}, c) J_{SC}, and d) FF of control and target devices treated with different process based on 20 cells.

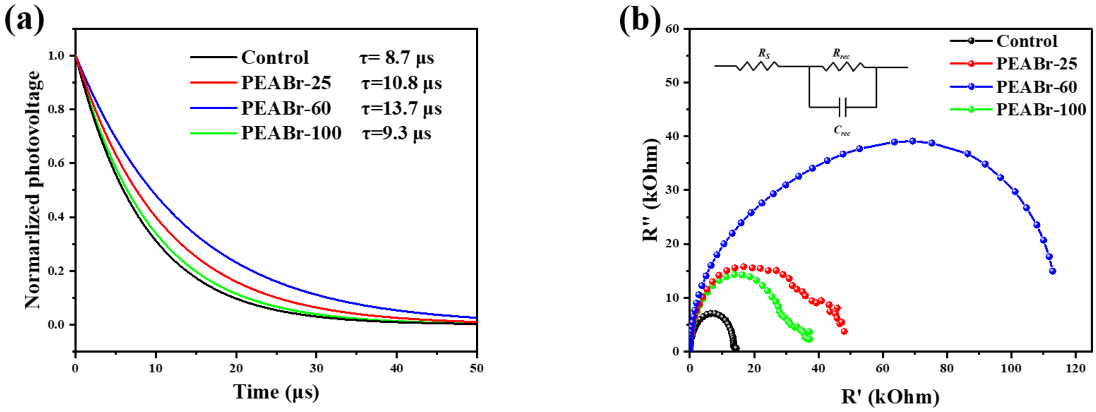

Figure S9. a) TPV curves and b) Nyquist plots for the control and target devices with different post-treatment process.

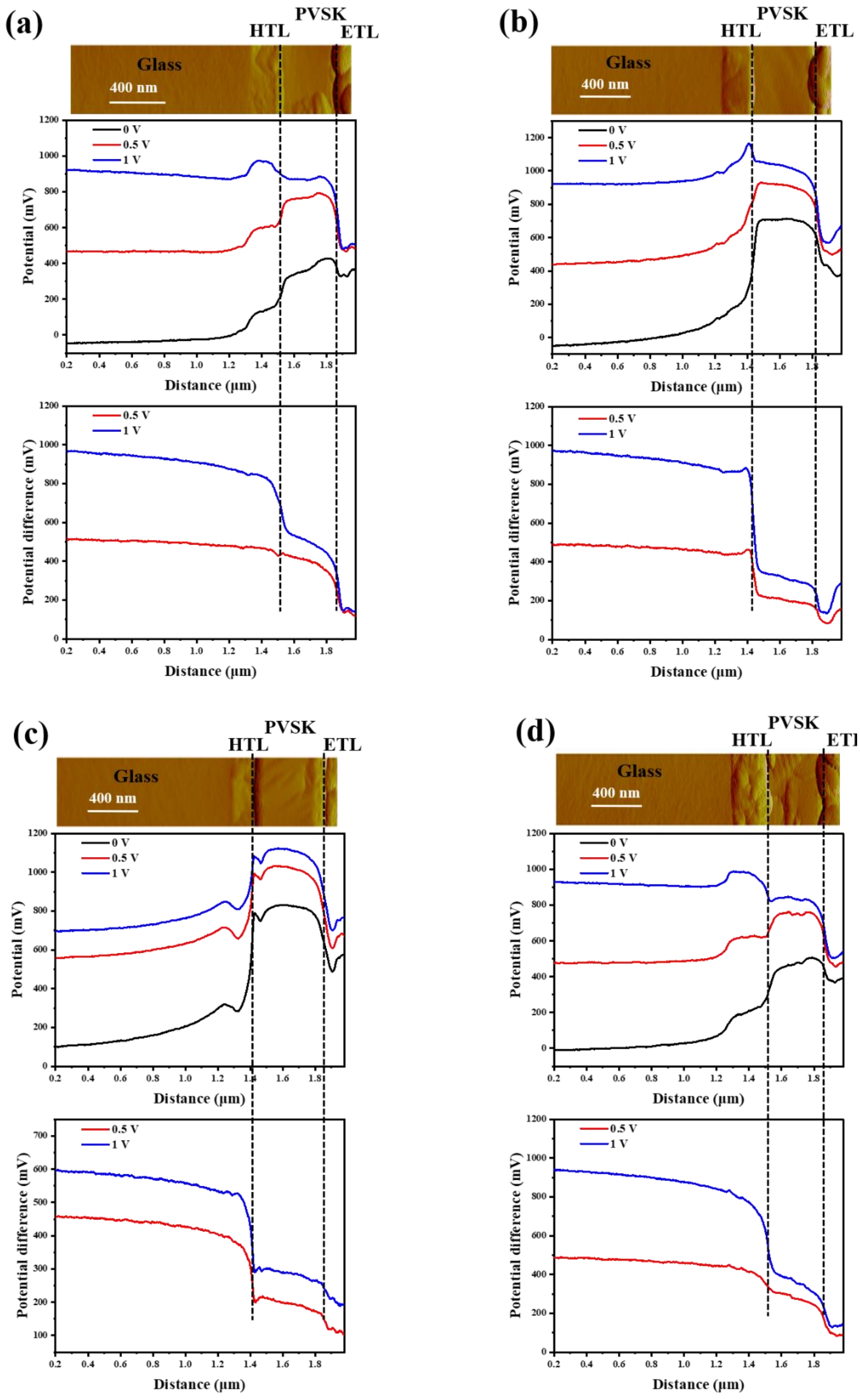

Figure S10. Cross-sectional KPFM potential maps and the corresponding potential and potential difference profiles of a) control, b) PEABr-25, c) PEABr-60, and d) PEABr-100 PSCs.

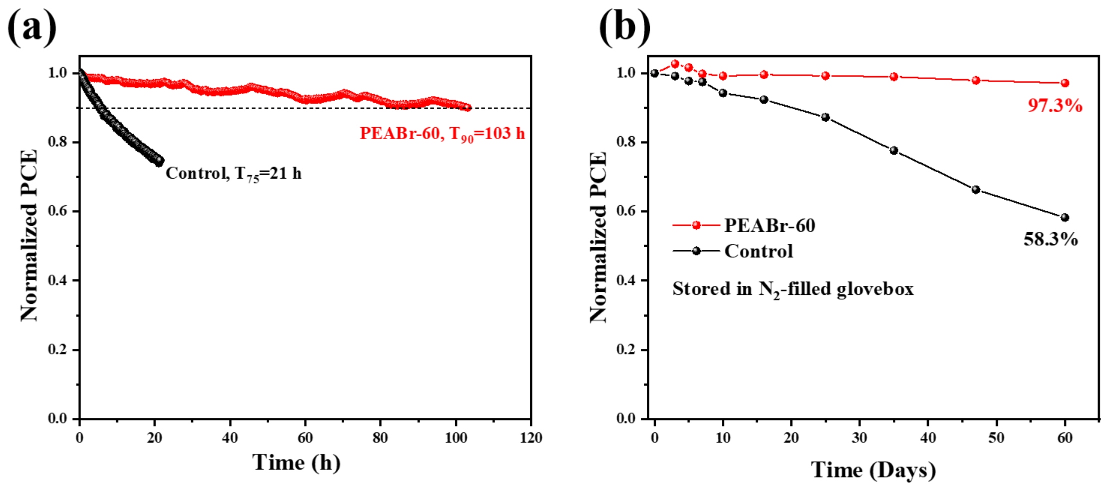

Figure S11. a) Continuous MPP tracking of encapsulated PEABr-60 and control device measured in the air under white LED illumination. b) Storage stability of PEABr-60 and control devices for 60 days.

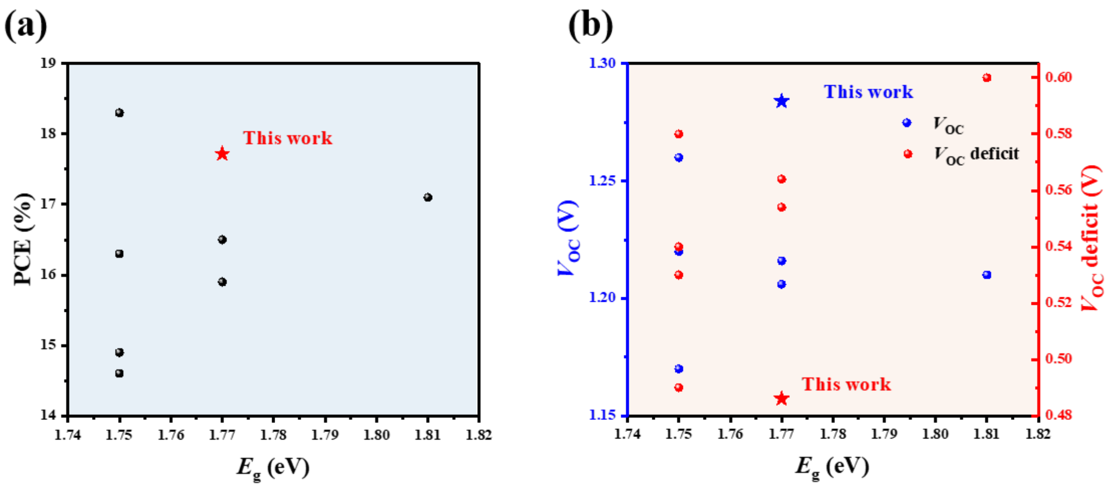

Figure S12. PCE and V_{OC} as a function of E_g for the best inverted WBG ($E_g \geq 1.75$ eV) PSCs reported in literature in comparison with our work.

Table S1. TRPL fitting data of the control and PEABr-treated perovskite films.

**Films on bare glass**	**τ_1 (ns)**	**A_1 (%)**	**τ_2 (ns)**	**A_2 (%)**	**τ_{avg} (ns)**
Control	103.5	20.27	470	79.73	449.46
PEABr-25	206.1	16.69	650.7	83.31	622.12
PEABr-60	222.6	17.13	690.8	82.87	659.18
PEABr-100	190.9	19.64	583.6	80.36	551.75

Table S2. TRPL fitting data of the control and PEABr-treated perovskite films with C_{60} coated.

**Films with C_{60}**	**τ_1 (ns)**	**A_1 (%)**	**τ_2 (ns)**	**A_2 (%)**	**τ_{avg} (ns)**
Control	1.56	41.11	5.58	58.89	4.92
PEABr-25	10	41.57	23.52	58.43	20.38
PEABr-60	5.75	65.84	27.21	34.16	21.00
PEABr-100	3.54	56.74	12.96	43.26	10.48

Table S3. Summary on main photovoltaic parameters of control and target PSCs treated with different annealing process. The data are obtained from 20 cells for control and target devices.

Samples	V_{OC} (V)	J_{SC} (mA·cm^{-2})	FF (%)	PCE (%)
Control	1.182 ± 0.01	17.25 ± 0.07	76.69 ± 0.88	15.64 ± 0.24
(champion device)	(1.186)	(17.29)	(78.26)	(16.06)
PEABr-25	1.250 ± 0.01	17.12 ± 0.09	78.04 ± 0.91	16.69 ± 0.24
(champion device)	(1.255)	(17.16)	(79.41)	(17.10)
PEABr-60	1.277 ± 0.01	17.18 ± 0.09	78.91 ± 1.04	17.31 ± 0.26
(champion device)	(1.284)	(17.20)	(80.29)	(17.74)
PEABr-100	1.204 ± 0.01	17.04 ± 0.09	77.15 ± 1.30	15.83 ± 0.26
(champion device)	(1.207)	(17.11)	(79.25)	(16.37)

Table S4. Photovoltaic performance metrics of state-of-the-art organic-inorganic hybrid inverted wide-bandgap (1.75 ~ 1.8 eV) PSCs.

Year	Type	E_g (eV)	V_{OC} deficit (V)	V_{OC} (V)	FF (%)	J_{SC} ($mA·cm^2$)	PCE (%)	Ref.
2016	p-i-n	1.75	0.54	1.21	77.9	15.8	14.9	[2]
2018	p-i-n	1.75	0.53	1.22	73.2	16.3	14.6	[3]
2018	p-i-n	1.75	0.58	1.17	80	17.5	16.3	[4]
2019	p-i-n	1.81	0.6	1.21	79.5	17.8	17.1	[5]
2019	p-i-n	1.77	0.554	1.216	79.7	17	16.5	[6]
2020	p-i-n	1.77	0.564	1.206	77	17.1	15.9	[7]
2020	p-i-n	1.75	0.49	1.26	80	18.12	18.3	[8]
	**p-i-n**	**1.77**	**0.486**	**1.284**	**80.3**	**17.2**	**17.72**	**This work**

Table S5. Summary on main photovoltaic parameters of semi-transparent WBG, filtered LBG, and perovskite/perovskite 4-T tandem PSC.

Sample	V_{OC} (V)	J_{SC} (mA cm^{-2})	FF (%)	PCE (%)
Original LBG PSC	0.857	30.9	80.3	21.26
Filtered LBG PSC	0.831	13.5	81.8	9.17
WBG PSC	1.274	15.9	78.8	16.00
4-T tandem cell	-	-	-	25.17

References

[1] L. Krückemeier, U. Rau, M. Stolterfoht, T. Kirchartz, *Adv. Energy Mater.* **2020**, 10, 1902573.

[2] M. Hu, C. Bi, Y. Yuan, Y. Bai, J. Huang, *Adv. Sci.* **2016**, 3, 1500301

[3] R. J. Stoddard, A. Rajagopal, R. L. Palmer, I. L. Braly, A. K. Y. Jen, H. W. Hillhouse, *ACS Energy Lett.* **2018**, 3, 1261.

[4] K. A. Bush, K. Frohna, R. Prasanna, R. E. Beal, T. Leijtens, S. A. Swifter, M. D. McGehee, *ACS Energy Lett.* **2018**, 3, 428.

[5] Y. M. Xie, Z. Zeng, X. Xu, C. Ma, Y. Ma, M. Li, C. S. Lee, S. W. Tsang, *Small* **2020**, 16, 1907226.

[6] R. Lin, K. Xiao, Z. Qin, Q. Han, C. Zhang, M. Wei, M. I. Saidaminov, Y. Gao, J. Xu, M. Xiao, A. Li, J. Zhu, E. H. Sargent, H. Tan, *Nat. Energy* **2019**, 4, 864.

[7] K. Xiao, R. Lin, Q. Han, Y. Hou, Z. Qin, H. T. Nguyen, J. Wen, M. Wei, V. Yeddu, M. I. Saidaminov, Y. Gao, X. Luo, Y. Wang, H. Gao, C. Zhang, J. Xu, J. Zhu, E. H. Sargent, H. Tan, *Nat. Energy* **2020**, 5, 870.

[8] Z. Li, J. Zhang, S. Wu, X. Deng, F. Li, D. Liu, C. C. Lee, F. Lin, D. Lei, C.-C. Chueh, Z. Zhu, A. K. Y. Jen, *Nano Energy* **2020**, 78, 105377.
